# An electroporation protocol for efficient DNA transfection in PC12 cells

**DOI:** 10.1007/s10616-013-9608-9

**Published:** 2013-07-12

**Authors:** Giuseppina Covello, Kavitha Siva, Valentina Adami, Michela A. Denti

**Affiliations:** 1 Laboratory of RNA Biology and Biotechnology, Centre for Integrative Biology (CIBIO), University of Trento, via delle Regole 101, 38123 Trento, Italy; 2 High-Throughput Screening Facility, Centre for Integrative Biology (CIBIO), University of Trento, via delle Regole 101, 38123 Trento, Italy; 3 CNR Institute of Neuroscience, Padua, Italy

**Keywords:** PC12 cells, Cell culture, DNA transfection, DNA electroporation, NGF, Neural differentiation

## Abstract

A wide variety of mammalian cell types is used in gene transfection studies. Establishing transfection methods that enable highly efficient DNA uptake has become increasingly important. PC12 is an established rat pheochromocytoma cell line, which responds to exposure to NGF with cessation of growth, expression of cytoplasmic processes, and differentiation into cells resembling sympathetic neurons. Although PC12 cells represent an important model system to study a variety of neuronal functions, they proved relatively difficult to transfect. We have compared the efficiency of three different chemical transfection reagents (Lipofectamine 2000, Lipofectamine LTX and TransIT-LT1) and of two electroporation systems (Neon and Gene Pulser Xcell) in transiently transfecting undifferentiated PC12 cells. By comparing efficiencies from replicate experiments we proved electroporation (in particular Neon) to be the method of choice. By optimizing different parameters (voltage, pulse width and number of pulses) we reached high efficiency of transfection (90 %) and viability (99 %). We also demonstrated that, upon electroporation, cells are not altered by the transfection and maintain their ability to differentiate.

## Introduction

PC12 cells are a cell line originating from pheochromocytoma in the rat adrenal medulla (Schaefer et al. 1987) and grow in culture as undifferentiated neuroblasts. Since its characterization in 1976 (Greene and Tischler 1976 **)** PC12 cells have become a commonly employed model system for studies of neuronal development and function (Grau and Greene 2012). One of the important features of PC12 cells is that they are small cells with a limited amount of cytoplasm and long doubling time. They possess remarkable ability to respond to nerve growth factor (NGF), a neurotrophic polypeptide, inducing morphological and biochemical changes resulting in differentiation of PC12 cells into a sympathetic neuron-like phenotype (Greene and Tischler 1976; Grau and Greene 2012; Nagase et al. 2005; Dhar et al. 2007; Park et al. 2007). For this reason PC12 cells have been regarded as a research model to study neuronal development, sympathetic neurotransmission and neurodegenerative diseases (Wang et al. 2002; Seth et al. 2002). In general, when challenged with physiological levels of NGF, these cells cease division, become electrically excitable, extend long branching neurites, and gradually acquire many characteristics of mature sympathetic neurons. Under serum-free conditions, NGF promotes not only neuronal differentiation of PC12 cells, but also their survival (Greene 1978; Fujita et al. 1989; Rukenstein et al. 1991). Several of their attributes have led to their widespread popularity in neurobiological research. These include their relatively high degree of differentiation before and after NGF treatment, homogeneous response to stimuli, availability in large numbers for biochemical studies, and suitability for genetic manipulations. However, finding transfection techniques that enable efficient DNA uptake into PC12 cells is relatively difficult. Moreover, it is important to have a good method to transfect these cells with high efficiency devoid of cellular alteration, and maintaining their ability to differentiate. An important limitation of working with PC12 cells is that they tend to be very sensitive to physical stress, alterations in temperature, pH shifts, or changes in osmolarity. Therefore, handling and manipulation during transfection is a crucial step.

Various transfection methods have been attempted to transfect this cell line. In general, cells can be gene-modified in vitro and in vivo using chemical or physical methods (Azzam and Domb 2004; Marples and Dachs 2002). Chemical methods of transfection are widely used, as they are relatively simple, cheap, and safe (Douglas 2008). They include calcium phosphate, liposomes, cationic lipids (e.g., dioleoyl trimethylammonium propane (DOTAP)), cationic polymers (e.g., polyethylenimine (PEI), dendrimers) and cationic polysaccharides (Eliyahu et al. 2005; Godbey et al. 1999a, b). In general, these reagents act via packaging mechanism to condense and deliver DNA to the cytoplasm of cells, usually by endocytosis (Vijayanathan et al. 2002). These reagents are used rapidly in high-throughput assays and can transfer DNA of various sizes (Ewert et al. 2008). However, they can be susceptible to nuclease degradation, are potentially harmful (Colombo et al. 2001) and are usually, but not always, cell cycle-dependent (Brunner et al. 2002).

In the particular case of PC12 cells, cationic lipids formulations have been employed to increase transfection efficiency. Using Lipofectamine 2000 (Invitrogen) the transfection efficiency was about 14 % and was similar to the efficiency obtained with polyethyleneimine (PEI) (15 %) (Lee et al. 2008). A higher efficiency (30 %) was reported with Metafectene Pro (Biontex) (Cogli et al. 2010). An increase in the transfection efficiency (40–50 %) was observed by simultaneous treatment with Lipofectamine and 0.1 μM GALA, a pH-sensitive fusogenic peptide which accelerates the endosomal escape of the plasmid/liposome complexes to the cytosol (Futaki et al. 2005). However, this method has not encountered wide popularity thereafter.

Physical methods, including electroporation, biolistics and injection, are used with varying success and are cell cycle-independent but may be more toxic for some cell types and usually require cell suspensions in vitro and specialized equipment (Villemejane and Mir 2009). However, the electroporation methods enable efficient transfer of exogenous DNA to a large number of cells and serve ideal in terms of material and time consumption. As with chemical methods of transfection, high-efficiency electroporation protocols for PC12 cells are not available. The literature reports efficiencies between 10 and 20 % (Darchen et al. 1995; Akamatsu et al. 1999) and 35 % (Lombardi et al. 2001), which increases to 50 % when parameters are carefully optimized (Espinet et al. 2000).

The aim of this work was to find a protocol ensuring high transfection efficiency in PC12 cells, while retaining viability and ability to differentiate. We compared two electroporation systems (Neon transfection and Gene Pulser Xcell) and three chemical transfection methods (Lipofectamine 2000, Lipofectamine LTX, TransIT-LT1).

## Materials and methods

### Plasmid

DNA plasmid used for transfection or electroporation was pEGFP-C1 (BD Biosciences Clontech, Palo Alto, CA, USA) driving the expression of an enhanced green fluorescent protein (EGFP) under the control of the CMV promoter. Plasmid was amplified in *Escherichia coli* DH5α; it was isolated and purified using Endo-Maxi Free Kit from QIAGEN. DNA purity and integrity were determined spectroscopically (OD_260nm_/OD_280nm_ = 1.90–2.00), (OD_260nm_/OD_230nm_ > 2.00).

### Culture of PC12 cells

Rat PC12 cells (ATCC entry CRL-1721) were grown at 37 °C (5 % CO_2_) in supplemented DMEM: Dulbecco’s modified Eagle’s medium with 4.5 % glucose (Lonza, Visp, Switzerland) supplemented with 10 % fetal bovine serum (Gibco, Grand Island, NY, USA), 5 % horse serum (Gibco), 1 mM glutamine (Gibco), 1 mM Penicillin/Streptomycin (Gibco). Cells were seeded onto T-75 cm^2^ flasks (Corning, NY, USA) coated with 50 ng/ml poly-d-lysine hydrobromide (Sigma, St. Louis, MO, USA), to achieve 70 % confluence. Cells from passages 8–10 were used. Cells were split every other day at a ratio of about 2:3. A Pasteur pipette was used to de-aggregate cell clusters.

### Viable cells counts

A Trypan Blue Stain exclusion test (Invitrogen, Carlsbad, CA, USA) was used to distinguish viable from nonviable cells. The suspended cells (9 μl) were mixed with 1 μl of 0.4 % Trypan Blue Stain and analyzed in the Countess™ Automated Cell Counter (Invitrogen) chamber slide. The percentage of viable cells was calculated as follows: (number of viable cells/total number of cells) × 100.

### Lipid-mediated DNA transfection

The transfection reagents used in this study were: TransIT-LT1 Transfection Reagent (Mirus, Madison, WI, USA), Lipofectamine 2000 and Lipofectamine LTX (Invitrogen).

4 × 10^4^ cells/well were seeded on poly-d-lysine-coated 24-well plates (Corning) one day before transfection and grown in supplemented DMEM at 37 °C and 5 % CO_2_. PC12 cells grown to 70 % confluence were transfected with the mammalian expression vector pEGFP-C1. Transfection procedures were performed as indicated by manufacturer. The ratio DNA (μg) : transfection reagent (μl) was always 1:3.

### Transfection by electroporation

DNA electroporation was performed with the Neon^®^ Transfection System MPK5000 (Invitrogen) or the Gene Pulser Xcell System (BIORAD, Hercules, CA, USA).

For the electroporation with the Neon System, PC12 cells were grown to 70 % confluence in a poly-d-lysine-coated T-25 flask and washed twice with 10 ml Phosphate-Buffered Saline without Ca^2+^ and Mg^2+^(PBS) (Lonza). This was followed by addition of 1 ml 1× trypsin (Lonza) and incubation for 2 min at 37 °C. After adding 9 ml of supplemented DMEM, the cells were resuspended, transferred in a 15 ml polypropylene tube (Sarstedt, Verona, Italy) and centrifuged at 2.200×*g* for 10 min. The pellet was resuspended in 10 ml of PBS and cells were counted in a Bürker chamber (PAUL MARIENFELD GmbH). Cells were pelleted again and re-suspended in Resuspension Buffer R (Neon 10 μl kit Invitrogen) to a final concentration of 1 × 10^7^ cells ml^−1^. 0.6 × 10^5^ and 1 × 10^5^ cells were transferred to a sterile 1.5 ml microcentrifuge tube (Sarstedt), brought to 10 μl final volume of cell suspension with Buffer R, and mixed with 500 ng of pEGFP-C1 vector. To optimize the best condition of transfection, electroporation was then carried out with different voltage, pulse and time parameters, according to manufacturer’s instructions, as reported in Results and Discussion. Cells were seeded in a 24- well poly-d-lysine-coated cell plate with 0.5 ml of pre-warmed supplemented DMEM without antibiotics.

For the electroporation with the Gene Pulser Xcell System, PC12 cells were grown to 70 % confluence in a poly-d-lysine-coated T-75 flask and washed and trypsinized as described above. After adding supplemented DMEM, the cells were resuspended, transferred in a polypropylene tube and centrifuged at 400×*g* for 5 min. The pellet was resuspended and counted as above, and re-suspended in an appropriate amount of PBS. 0.4 ml of cell suspension containing 6 × 10^5^ or 1 × 10^6^ cells and 8 μg of DNA (pEGFP-C1 vector), were incubated on ice for 10 min, and transferred into a 4-mm electroporation cuvette (BTX). The Gene Pulser Xcell System (BIORAD) was used for single-cuvette electroporation. Electroporations were carried out with different voltage and capacitance parameters, according to manufacturer’s instructions, as reported in Results and Discussion. Cells were seeded in a 12- well poly-d-lysine-coated cell plate with 1 ml of pre-warmed supplemented DMEM without antibiotics.

### High-content image acquisition and analysis

Upon chemical transfection or electroporation of pEGFP-C1 vector, EGFP expression and cell nuclei were visualized using Operetta High Content Imaging System (PERKIN ELMER, Monza, Italy). To count total cell numbers, nuclei were counterstained with Hoechst 33342 (Invitrogen). Forty-eight hours after transfection, cells were washed once with PBS and incubated in 0.5 ml of supplemented DMEM containing 1 mg/ml of Hoechst 33342, for 20 min at 37 °C and 5 % CO_2_. Cells were washed once with PBS and replaced with supplemented DMEM without phenol red (Gibco) for imaging. Images were acquired on an Operetta System using a 20× LWD objective in wide-field mode in combination with filters for Hoechst 33342 (excitation filter: 360–400 nm; emission filter: 410–480 nm) and Alexa Fluor 488 (excitation filter: 460–490 nm; emission filter: 500–550 nm). The laser autofocus was applied and 10 image fields were acquired per well. For quantitative analyses, individual cells were segmented based on the Hoechst 33342 nuclear stain using the Find Nuclei building block in the Harmony^®^ High Content Imaging and Analysis Software (PERKIN ELMER) and GFP intensity was quantified within the Hoechst-defined boundaries for each cell. The Select Population module of Harmony allowed to set a fluorescence intensity threshold in order to identify the sub-population of transfected cells and to determine the transfection efficiency. The average and standard error mean (SEM) were calculated from biological experimental triplicates and technical duplicates.

### Cell differentiation

Electroporated PC12 cells were plated at a density of 1 × 10^5^ cells/well on poly-d-Lysine-coated 12-well plates and grown in supplemented DMEM without antibiotics at 37 °C in 5 % CO_2_. After 24 h the medium was replaced with differentiation medium (Dulbecco’s modified Eagle’s medium with 4.5 % glucose supplemented with 0.3 % fetal bovine serum, 0.7 % horse serum, 1 mM glutamine) containing 75 ng/ml of NGF 2.5S (Invitrogen). The cells were maintained at 37 °C and 5 % CO_2_ and the differentiation medium was replenished every 2nd day for 7 days.

### Neurite analysis

Neuronal differentiation was estimated every day, for 7 days, after exposure to NGF 2.5S, by measurement of morphological parameters. Images were acquired using a fluorescence microscope (DFC420C, Leica, Milan, Italy) and a 20X objective (magnification). Filter A (Exciter BP340–380 nm; Dichromatic Mirror 400 nm) and I3 (Exciter BP450–490 nm; Dichromatic Mirror 510 nm) were used and the images were analysed with ImageJ software (http://imagej.nih.gov/ij/) to determine the neurite length (ImageJ plugin NeuronJ) and to quantify the percentage of differentiated cells. A neurite was defined as a process of length equal to or greater than one time the diameter of the cell body. Triplicate wells were used routinely for each experimental condition. Images of three different fields were taken per well. The experiment was repeated two times.

### Statistical analysis

All statistical analyses were performed using Graphpad Prism software package (GraphPad Software, San Diego, CA, USA). Student’s *t* test and One-way analysis of variance (ANOVA) with Bonferroni’s multiple comparison were adopted. A single level of 0.05 (*p < 0.05) was used, unless otherwise stated. Data are shown with the standard error of the mean (mean ± SEM, n = 3).

## Results and discussion

### Comparison of transfection chemical methods in PC12 cells

We compared transfection efficiencies obtained in PC12 cells with the lipopolyplex transfection reagent TransIT-LT1 (Mirus) and the cationic lipids Lipofectamine 2000 and LTX (Invitrogen). Cells were grown in conditions promoting proliferation including use of an enriched medium, but the transfections were carried out in a serum- and antibiotic- free environment. We performed these experiments with different amounts of pEGFP-C1 DNA (Clontech), a plasmid driving the expression of an enhanced green fluorescent protein (EGFP) under the control of the CMV promoter. The ratio DNA (μg): transfection reagent (μl) was 1:3 and we transfected increasing amounts of plasmid (0.25, 0.5, 0.75 and 1 μg).

**Fig. 1:**
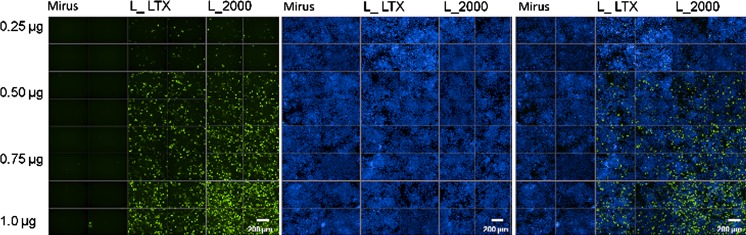
Transfection of rat PC12 cells. PC12 cells were transfected by liposoluble agents (TransIT-LT1 Transfection Reagent [Mirus], Lipofectamine LTX [L_LTX] and Lipofectamine 2000 [L_2000]) and different concentrations of a plasmid encoding EGFP. Fluorescence images of transfected PC12 cells were analyzed by High-Content screening system (Operetta) 48 h after transfection. The panel shows photographs taken with a *green filter* (*left*) and a *blue filter* (*center*), and the merged images (*right*). Original magnification ×20 (*Scale bars* = 200 μm). (Color figure online)

Forty-eight hours after transfection, nuclei were stained with the viable Hoechst 33342 fluorescent dye and images were acquired on an Operetta System, which combines fluorescence microscopy in a multi-well format with automated image acquisition and quantitative analysis (Fig. 1). Data analysis for transfection efficiency was performed by using the Harmony^®^ High-Content Imaging Software (Perkin Elmer), comparing the number of EGFP-positive cells (detected with an Alexa Fluor 488 filter) to the total number of cells (detected with an Hoechst 33342 filter). Mock transfection controls using the transfection reagent without DNA had an auto-fluorescence background in both the Hoechst 33342 or Alexa Fluor 488 channels comparable to un-transfected PC12 cells (data not shown).

**Fig. 2:**
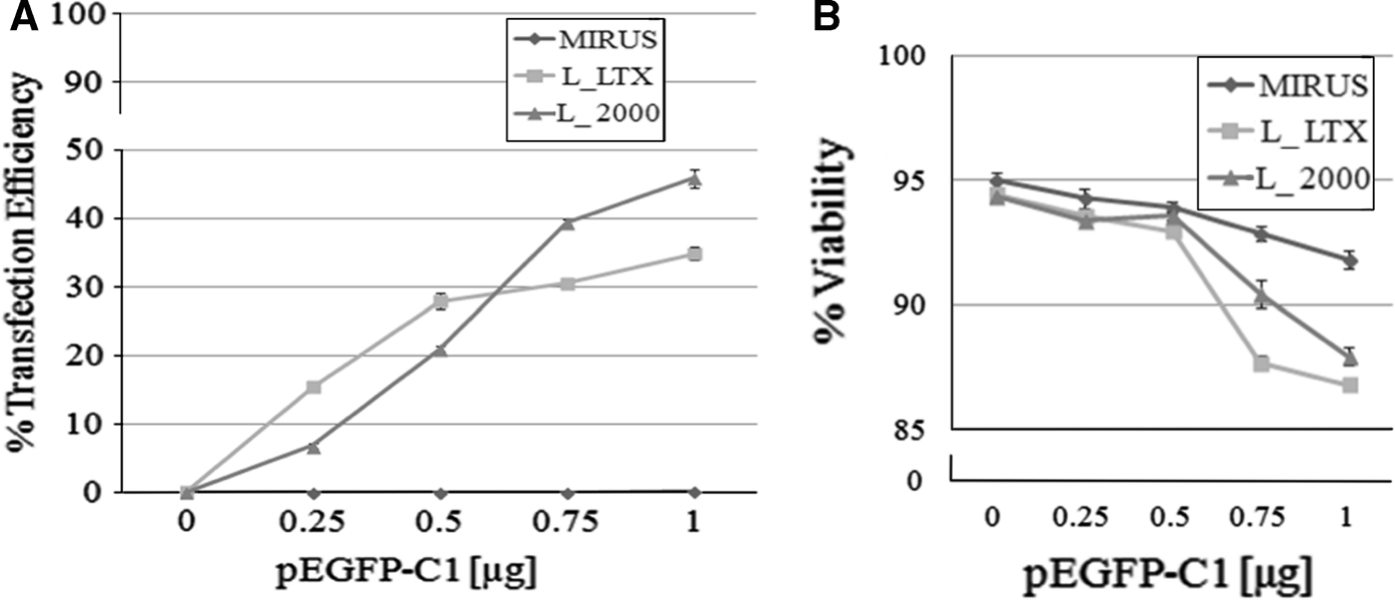
Transfection efficiency and viability of rat PC12 cells. **a** The percent of transfected cells was calculated by dividing the number of EGFP positive cells by the total population. The best result was obtained by using 1 μg of plasmid and Lipofectamine 2000 (efficiency of transfection 45.9 %). **b** The percentage of viable cells after transfection was measured by a Trypan Blue assay. The best cell viability (93.6 %) was obtained with 0.5 μg of plasmid and Lipofectamine 2000. Data represent mean ± SEM obtained from triplicates

As shown in Fig. 2a, the TransIT-LT1 Transfection Reagent (Mirus) was not effective in facilitating transfection of DNA in PC12 cells, even when a higher concentration of DNA was used. However, in parallel experiments performed on the HEY4 ovarian cancer cell line, TransIT-LT1 had a transfection efficiency of approximately 35 %, thereby indicating that efficiency depends on the cell type (data not shown).

On the contrary, when we performed the transfection experiments by using Lipofectamine LTX and Lipofectamine 2000 we managed to transfect DNA into PC12 cells, with efficiencies that ranged from 7 and 15 % respectively, with 0.25 μg DNA, to 35 and 46 % respectively, when 1 μg of DNA was used.

As expected, transfection efficiency for both reagents correlates with the amounts of DNA used. However, in comparing the transfection efficiency of the two cationic lipids, Lipofectamine LTX seems to perform better than Lipofectamine 2000 at low DNA amounts, while Lipofectamine 2000 outperforms Lipofectamine LTX at higher DNA amounts.

In particular, comparing our results with those by Lee and colleagues (2008), with Lipofectamine 2000 we reached 21 % transfection efficiency (with 0.5 μg of DNA in a 24-well plate) while Lee and collaborators reported 14 % efficiency in similar conditions (1 μg of DNA in a 12-well plate).

Cell viability was measured by Trypan Blue Staining 48 h after transfection (Fig. 2b). Mock-transfected cells had a viability comparable to the non-transfected cells with either of the three transfection reagents (approx. 95 %). For all methods, viability decreased as the DNA amounts increased. However, as clearly indicated in Fig. 2b, there is a variation of trend for different reagents. TransIT-LT1 had a milder impact on cell viability, reaching 92 % when 1 μg of DNA was used. On the contrary, Lipofectamine 2000 and Lipofectamine LTX reached 87–88 % viability in those conditions.

### DNA electroporation in PC12 cells

As the transfection efficiency obtained with Lipofectamine 2000 was not sufficient for our purposes, we investigated if we could obtain a higher percentage of transfected PC12 cells with an electroporation method (Neon Transfection System, Invitrogen).

To optimize conditions, 0.5 μg of plasmid DNA and two different cell densities (6 × 10^4^ or 1 × 10^5^ cells/well) were used. Moreover, a range of voltage, pulse width and pulse number combinations were tested (Table 1).

**Table 1:** Parameters for DNA electroporation by Neon transfection System, transfection efficiency and cell viability

Pulse voltage (V)	Pulse width (ms)	Pulse no.	Transfection Efficiency (%)		Cell viability (%)	
			0.6 × 10^5^ cells/well	1 × 10^5^ cells/well	0.6 × 10^5^ cells/well	1 × 10^5^ cells/well
Control without electroporation			–	–	90.00	90.00
1,500	20	1	20.33	93.94	63.30	86.70
1,400	30	1	64.37	80.97	38.40	67.70
1,500	10	3	66.20	90.37	54.70	99.00
1,400	10	3	84.63	87.07	64.20	79.70
1,300	30	1	84.37	97.83	86.70	62.87
1,000	40	1	91.32	94.03	24.90	51.70

**Fig. 3:**
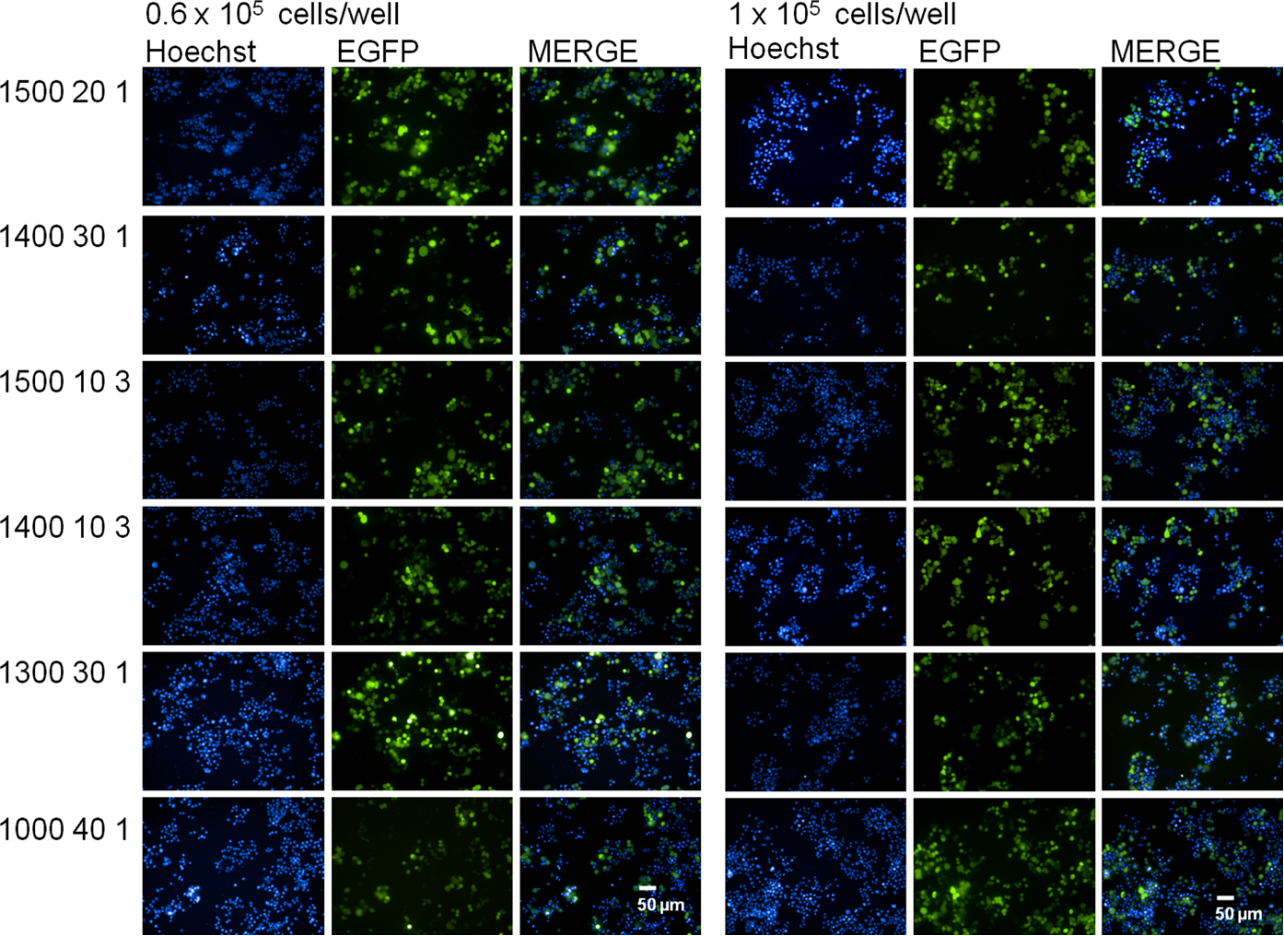
Representative images of PC12 cells 48 h after electroporation. Electroporation was carried out using different conditions of voltage (1000, 1300, 1400 or 1500 Volts), pulse width (10, 20, 30 or 40 ms) and pulse number (1 or 3 pulses), as indicated on the left side of the panels. Pictures were taken with the high-content operetta system with a *blue filter* (Hoechst) and a *green filter* (EGFP), and merged (MERGE). Original magnification: ×20 (*Scale bars* = 50 μm). (Color figure online)

**Fig. 4:**
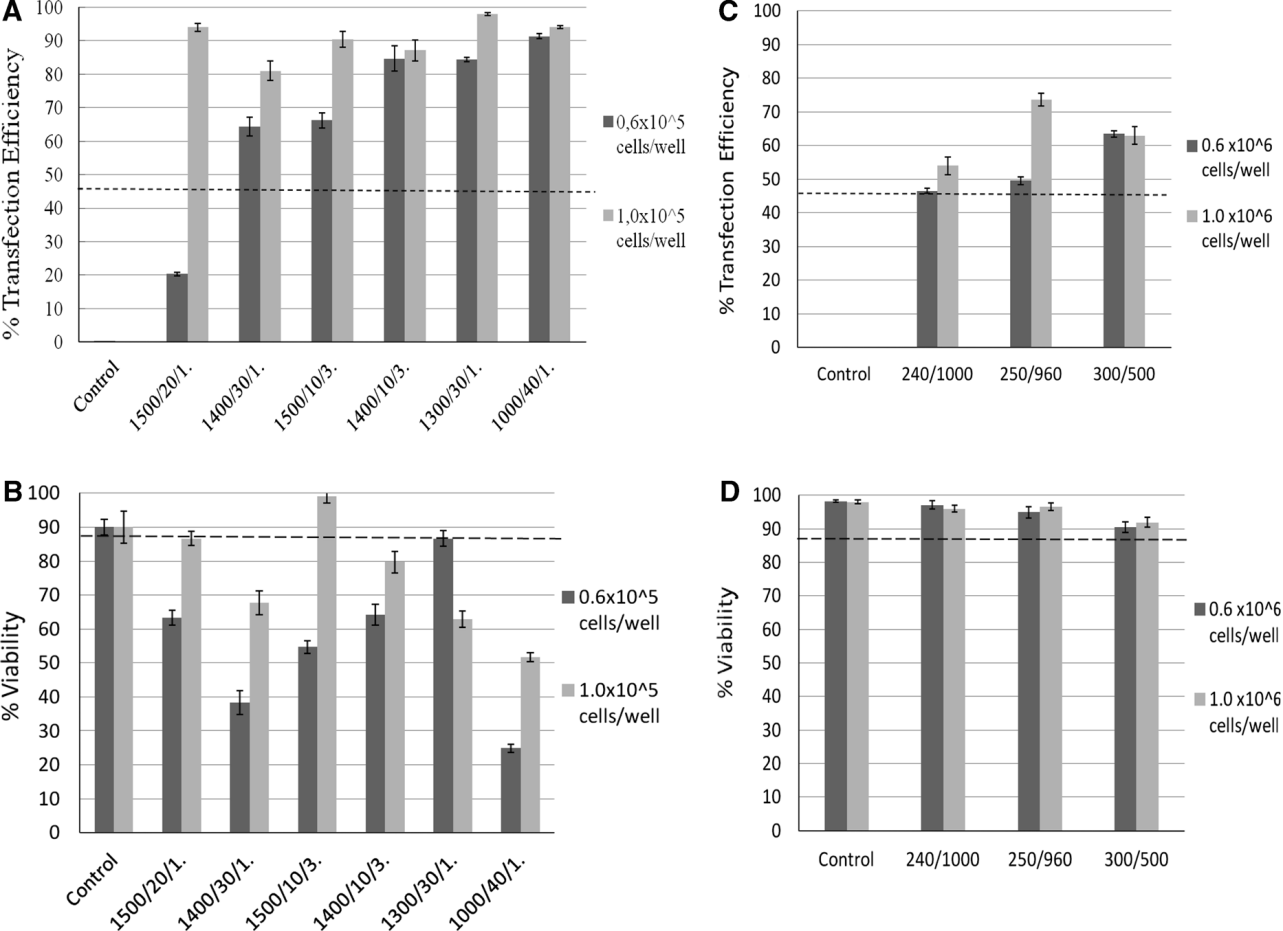
Optimization of PC12 cells transfection by electroporation. Electroporation was carried out in different conditions which are indicated on the panels' x-axes in the format "voltage (V)/pulse width (ms)/pulse number" for panels A and B, and in the format "pulse voltage (V)/capacitance (μF)" for panels C and D. **a** Histograms of percentages of transfected PC12 cells show an increase of efficiencies comparing Neon electroporation with lipofecting agents. *Dashed line* represents the best transfection efficiency obtained in lipofection experiments as shown in Fig. 2. The best condition (99 %) was obtained by 0.5 μg of plasmid, 1 × 10^5^ cells/well, and 1 pulse of 1,300 Volt and 30 ms. **b** The percentage of viable cells after Neon electroporation was measured by Trypan Blue Staining. The best cell viability (97.8 %) was obtained with 0.5 μg of plasmid, 1 × 10^5^ cells/well, and 3 pulses of 1,500 Volt and 10 ms each. *Dashed line* represents worst viability percentage obtained in lipofection experiments as shown in Fig. 2. **c** Transfection efficiency of electroporation experiments performed using Gene Pulser Xcell. *Dashed line* represents the best transfection efficiency obtained in lipofection experiments as shown in Fig. 2. **d** Viability of cells electroporated with Gene Pulser Xcell. *Dashed line* represents worst viability percentage obtained in lipofection experiments as shown in Fig. 2. Data represent mean ± SEM obtained from triplicates

Forty-eight hours after transfection, nuclei were stained with Hoechst 33342 and images were acquired on an Operetta System (Fig. 3). Data analysis of transfection efficiency was carried out as detailed above and revealed that the density of the cells in the suspension is one of the most important variables affecting transfection efficiency in our electroporation protocols (Fig. 4a). In fact, with higher cell density, any electroporation condition tested yields high transfection efficiency, ranging between 80 and 98 %. On the contrary, when a lower cell density was used, conditions can be separated into two classes, based on the effect they have on transfection efficiency: a high-efficiency class (84–91 %) and a low-efficiency class (20–66 %).

It has to be pointed out, however, that all conditions, except one (6 × 10^4^ cells, 1,500 V, 20 ms, 1 pulse), yielded a transfection efficiency higher than that one obtained by the use of Lipofectamine 2000 (dashed line in Fig. 4a).

The method shows high reproducibility, as determined by comparing efficiencies from several replicate experiments.

Cell viability was measured by Trypan Blue Staining 48 h after transfection (Table 1). Mock-electroporated cells were manipulated as the electroporated cells but did not receive DNA nor an electrical pulse, and had a viability comparable to the non-electroporated cells (approx. 90 %). In the case of cell viability, there was no clear correlation with cell density, although in general cells at higher density performed better (Fig. 4b). Furthermore, different electroporation conditions worked better for the different cell densities, and a general trend could not be inferred from the graph in Fig. 4b. In general, the electroporation protocol appeared to be more pernicious to PC12 cells than the lipofection protocols, as only few conditions provided a cell viability comparable or higher than that one obtained by Lipofectamine LTX and 1 μg of DNA (dashed line in Fig. 4b).

In order to establish if electroporation in general is a better method than lipofection, or if the Neon System in particular has a high performance in transfecting PC12 cells, we performed electroporation with a different system, namely Gene Pulser Xcell (Bio-Rad). 8 μg of plasmid DNA and two different cell densities (6 × 10^5^ or 1 × 10^6^ cells/well) were used. The increase in DNA and cell amounts, compared to Neon System, is due to the fact that, while the Neon system is miniaturized to use electroporation tips and 10 μl transfection volumes, the Gene Pulser XCell requires specific 400 μl cuvettes. For each cell density we tested three different voltage and capacitance combinations (Table 2), in triplicate experiments. Namely, we tried 240 V/1,000 μF (Murphy et al. 2008), 250 V/960 μF (Yaron et al. 2001) and 300 V/500 μF (Lombardi et al. 2001). Analysis of the transfection efficiency and cell viability was carried out as described above. As visible in Fig. 4c and in Table 2, in our hands the Gene Pulser Xcell system outperformed lipofection methods, reaching efficiencies higher than the ones reported in the literature. However, when comparing Gene Pulser Xcell with Neon System, the latter remained the method of choice for the transfection of PC12 cells (Fig. 4a, c). As far as viability is concerned, though, Gene Pulser Xcell compared to lipofection (Fig. 4d) and was better than Neon System in the majority of conditions.

**Table 2:** Electroporation parameters applied for DNA transfection by Gene Pulser Xcell, transfection efficiency and cell viability

Pulse voltage (V)	Capacitance (μF)	Transfection efficiency (%)		Cell viability (%)	
		0.6 × 10^6^ cells/well	1 × 10^6^ cells/well	0.6 × 10^6^ cells/well	1 × 10^6^ cells/well
Control without electroporation		–	–	98.22	97.94
240	1,000	46.54	54.01	97.08	95.98
250	960	49.56	73.58	94.88	96.56
300	500	63.44	62.98	90.42	91.91

For our purposes, the best electroporation conditions were obtained with Neon transfection System, 1 × 10^5^ cells/well and 3 pulses of 1,500 V and 10 ms each. These conditions, in fact, yielded 90 % transfection efficiency and 99 % viability. We have chosen these conditions for the subsequent experiments.

### Cell differentiation and neurite analysis

**Fig. 5:**
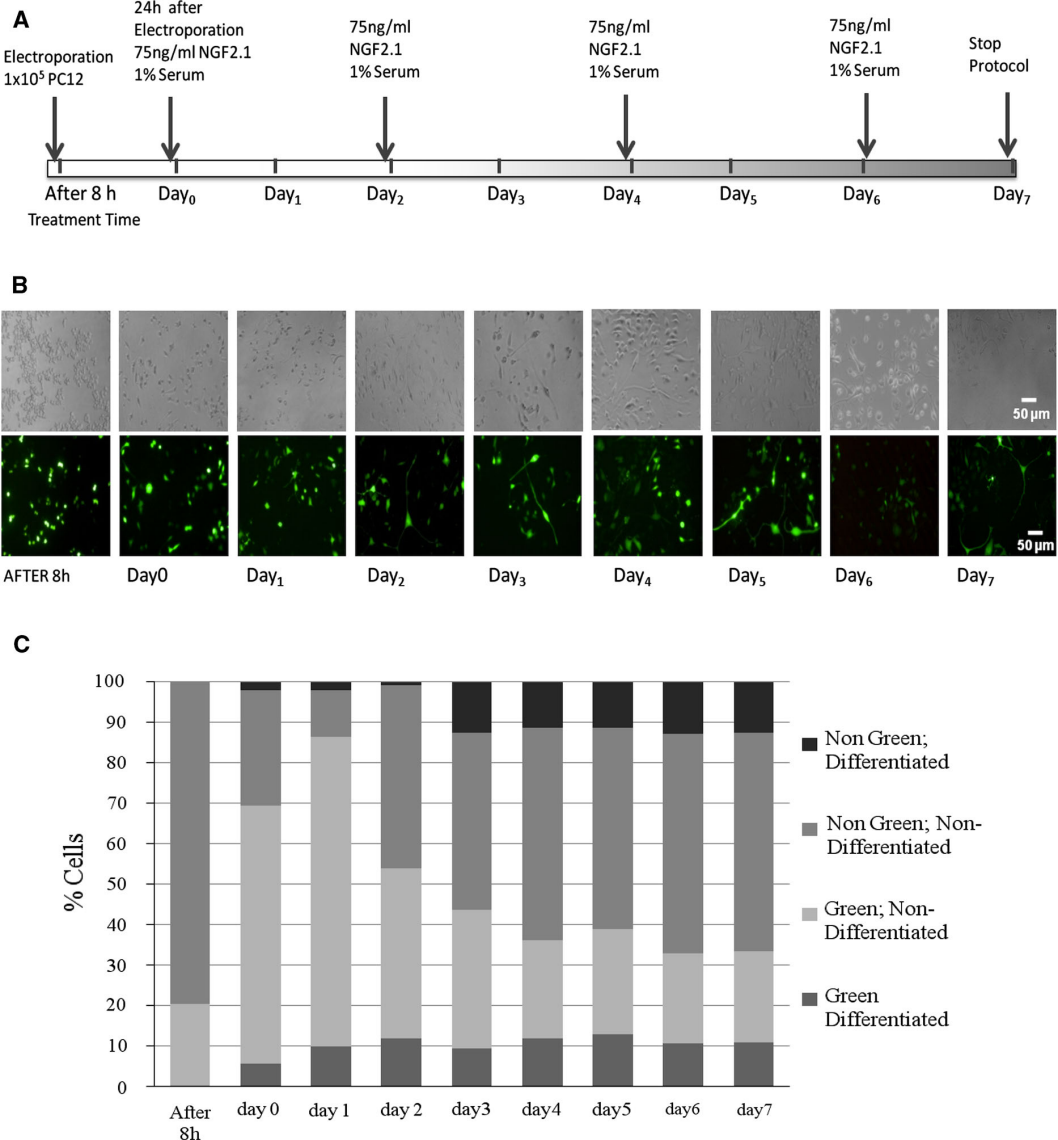
Differentiation of electroporated PC12 cells. **a** Time course of PC12 differentiation. At each indicated time point, pictures were taken in bright field and in *green* fluorescence, and cells were counted. **b** Representative images of PC12 cells treated with NGF for the indicated time. Original magnification: ×20 (*Scale bars* = 50 μm). **c** Histogram of cell numbers after transfection and differentiation. Cells were grouped in four categories: *green*, differentiated cells; *green*, non-differentiated cells; *non-green*, differentiated cells; *non-green*, non-differentiated cells. Data represent images of three different fields taken per each well. The experiment was repeated two times. (Color figure online)

With the aim of investigating whether the electroporated PC12 cells retain the ability to differentiate into neuron-like cells, we transfected the cells using the conditions reported above, and treated them with NGF 2.5S (75 ng/ml) starting 24 h after transfection (day 0) and for the subsequent 7 days (Fig. 5a).

Images were taken at 8 h and every 24 h after transfection, in bright field and with a green fluorescence filter (Fig. 5b). NGF-treated PC12 cells showed increase in neurite length and generation during time (Fig. 5b). The total number of cells and the number of cells presenting neurites was counted in the bright-field images. Differentiated PC12 cells progressively increased reaching 23 % of total cells 4 days after induction. At later times the percentage of differentiated cells remained stable (Fig. 5c).

EGFP expression was observed both in differentiated and non-differentiated cells. The intensity of EGFP signal increased over time. At the initial time point, the intracellular expression of EGFP in NGF-differentiated PC12 cells was located in the nucleus. At later time points, it was around cytoplasm and inside the nucleus. On the 7th day post-transfection, EGFP was seen throughout the entire cell, spreading out to the tips of the neurite extensions (not shown).

The number of transfected cells was counted in pictures taken with the GFP filter. Already 8 h after electroporation 20 % of cells showed EGFP signal. When NGF was added, 24 h after electroporation, 69 % of cells were EGFP-positive. The maximum transfection efficiency was reached 48 h after electroporation (86 %), and rapidly decreased to reach 33 % at days 6 and 7.

The discrepancy between the maximum transfection efficiency in these experiments and the results described in the previous paragraph (where 90 % of transfection efficiency was obtained) might reflect the change in serum percentages in the growth medium and the addition of NGF, 24 h after transfection.

Electroporation posed no impairment on differentiation ability of PC12 cells, since about half of the differentiated cells were green. On the contrary, our observations suggest that electroporation might even induce PC-12 cells to differentiate. It is remarkable in fact, that approximately 30 % of green cells were presenting neurite outgrowth, from day 4 onwards, while the percentage of differentiated cells in non-green PC-12 cells was about 20 %.

This stimulus to differentiate might explain why at days 0, 1 and 2, green differentiated cells were already present (6, 10 and 12 % of the total cells) while non-green differentiated cells reached comparable percentages (12 %) only at day 3.

Moreover, looking at the actual cell numbers, it appears that at day 2 non-green, non-differentiated cells started outnumbering green, non-differentiated cells, possibly indicating a higher proliferation rate of non-transfected cells, compared to non-transfected cells.

## References

[CR1] Akamatsu W, Okano HJ, Osumi N, Inoue T, Nakamura S, Sakakibara S-I, Miura M, Matsuo N, Darnell RB, Okano H (1999). Mammalian ELAV-like neuronal RNA-binding proteins HuB and HuC promote neuronal development in both the central and the peripheral nervous systems. Proc Natl Acad Sci USA.

[CR2] Azzam T, Domb AJ (2004). Current developments in gene transfection agents. Curr Drug Deliv.

[CR3] Brunner S, Furtbauer E, Sauer T, Kursa M, Wagner E (2002). Overcoming the nuclear barrier: cell cycle independent nonviral gene transfer with linear polyethylenimine or electroporation. Mol Ther.

[CR4] Cogli L, Progida C, Lecci R, Bramato R, Kruuttgen A, Bucci C (2010). CMT2B-associated Rab7 mutants inhibit neurite outgrowth. Acta Neuropathol.

[CR5] Colombo MG, Citti L, Basta G, De Caterina R, Biagini A, Rainaldi G (2001). Differential ability of human endothelial cells to internalize and express exogenous DNA. Cardiovasc Drugs Ther.

[CR6] Darchen F, Senyshyn J, Brondyk WH, Taatjes DJ, Holz RW, Henry J-P, Denizot J-P, Macara IG (1995). The GTPase Rba3a is associated with large dense core vesicles in bovine chromaffin cells and rat PC12 cells. J Cell Sci.

[CR7] Dhar S, McConnell MP, Gharibjanian NA, Young CM, Rogers JM, Nguyen TD, Evansl GR (2007). Herpes simplex virus-thymidine kinase-based suicide gene therapy as a molecular switch off for nerve growth factor production in vitro. Tissue Eng.

[CR8] Douglas KL (2008). Toward development of artificial viruses for gene therapy: a comparative evaluation of viral and non-viral transfection. Biotechnol Prog.

[CR9] Eliyahu H, Barenholz Y, Domb AJ (2005). Polymers for DNA delivery. Molecules.

[CR10] Espinet C, Gómez-Arbonés X, Egea J, Comella JX (2000). Combined use of the green and yellow fluorescent proteins and fluorescence-activated cell sorting to select populations of transiently transfected PC12 cells. J Neurosci Methods.

[CR11] Ewert KK, Ahmad A, Bouxsein NF, Evans HM, Safinya CR (2008). Non-viral gene delivery with cationic liposome-DNA complexes. Methods Mol Biol.

[CR12] Fujita K, Lazarovici P, Guroff G (1989). Regulation of the differentiation of PC12 pheochromocytoma cells. Environ Health Perspect.

[CR13] Futaki S, Masui Y, Nakase I, Sugiura Y, Nakamura T, Kogure K, Harashima H (2005). Unique features of a pH-sensitive fusogenic peptide that improves the transfection efficiency of cationic liposomes. J Gene Med.

[CR14] Godbey WT, Wu KK, Mikos AG (1999). Poly (ethylenimine) and its role in gene delivery. J Control Release.

[CR15] Godbey WT, Wu KK, Mikos AG (1999). Tracking the intracellular path of poly (ethylenimine)/DNA complexes for gene delivery. Proc Natl Acad Sci USA.

[CR16] Grau CM, Greene LA (2012). Use of PC12 cells and rat superior cervical ganglion sympathetic neurons as models for neuroprotective assays relevant to Parkinson’s disease. Methods Mol Biol.

[CR17] Greene LA (1978). Nerve growth factor prevents the death and stimulates the neuronal differentiation of clonal PC12 pheochromocytoma cells in serum-free medium. J Cell Biol.

[CR18] Greene LA, Tischler AS (1976). Establishment of a noradrenergic clonal line of rat adrenal pheochromocytoma cells which respond to nerve growth factor. Proc Natl Acid Sci USA.

[CR19] Lee JH, Ahn HH, Kim KS, Lee JY, Kim MS, Lee B, Khang G, Lee HB (2008). Polyethyleneimine-mediated gene delivery into rat pheochromocytoma PC-12 cells. J Tissue Eng Regen Med.

[CR20] Lombardi D, Palescandolo E, Giordano A, Paggi MG (2001). Interplay between the antimetastatic nm23 and the Retinoblastoma-related Rb2/130 genes in promoting neuronal differentiation of PC12 cells. Cell Death Diff.

[CR21] Marples B, Dachs GU (2002). Cancer gene therapy: “delivery, delivery, delivery”. Front Biosci.

[CR23] Murphy KL, Zhang X, Gainetdinov RR, Beaulieu J-M, Caron MG (2008). A regulatory domain in the N-terminus of tryptophan hydroxylase 2 controls enzyme expression. J Biol Chem.

[CR24] Nagase H, Yamakuni T, Matsuzaki K, Kasahara J, Hinohara Y, Kondo S, Mimaki Y, Sashida Y (2005). Mechanism of neurotrophic action of nobiletin in PC12D cells. Biochemistry.

[CR25] Park IK, Lasiene J, Chou SH, Horner PJ, Pun SH (2007). Neuron-specific delivery of nucleic acids mediated by Tet1-modified poly(ethylenimine). J Gene Med.

[CR26] Rukenstein A, Rydel R, Greene L (1991). Multiple agents rescue PC12 cells from serum-free cell death by translation- and transcription independent mechanisms. J Neurosci.

[CR27] Schaefer T, Karli UO, Scweizer FE, Burger MM (1987). Docking of chromaffin granules-a necessary step in exocytosis?. Biosci Rep.

[CR28] Seth K, Agrawal AK, Aziz MH, Ahmad A, Shukla Y, Mathur N, Seth PK (2002). Induced expression of early response genes/oxidative injury in rat Pheochromocytoma (PC12) cell line by 6-hydroxydopamine: implication for Parkinson’s disease. Neurosci Lett.

[CR29] Vijayanathan V, Thomas T, Thomas TJ (2002). DNA nonaparticles and development of DNA delivery vehicles for gene therapy. Biochemistry.

[CR30] Villemejane J, Mir LM (2009). Physical methods of nucleic acid transfer: general concepts and applications. Br J Pharmacol.

[CR31] Wang R, Zhou J, Tang XC (2002). Tacrine attenuates hydrogenperoxide-induced apoptosis by regulating expression of apoptosis-related genes in rat PC12 cells. Brain Res Mol Brain Res.

[CR32] Yaron Y, McAdara JK, Lynch M, Hughes E, Gasson JC (2001). Identification of novel functional regions important for the activity of HOXB7 in mammalian cells. J Immunol.

